# Introduction

**DOI:** 10.1007/978-3-030-57081-1_1

**Published:** 2020-10-14

**Authors:** Anita Hardon

**Affiliations:** University of Amsterdam, Amsterdam, Noord-Holland the Netherlands

## Abstract

The everyday lives of contemporary youth are awash with chemicals to boost pleasure, energy, sexual performance, appearance, and health. What do pills, drinks, sprays, powders, and lotions do for youth? What effects are youth seeking? The ChemicalYouth ethnographies presented here, based on more than five years of fieldwork conducted in Amsterdam, Brooklyn, Cayagan de Oro, Paris, Makassar, Puerto Princesa, and Yogyakarta, show that young people try out chemicals together, compare experiences, and engage in collaborative experiments. *ChemicalYouth: Navigating Uncertainty: In Search of the Good Life* makes a case for examining a broader range of chemicals that young people use in their everyday lives. It focuses not just on psychoactive substances—the use of which is viewed with concern by parents, educators, and policymakers—but all the other chemicals that young people use to boost pleasure, moods, vitality, appearance, and health, purposes for using chemicals that have received far less scholarly attention. It takes the use of chemicals as *situated practices* that are embedded in social relations and that generate shared understandings of efficacy. More specifically, it seeks to answer the question: how do young people balance the benefits and harms of chemicals in their quest for a good life?

My friend Hannah is worried. Her 18-year-old son Fedde is traveling through Australia, as some young people do after completing high school, and he is broke. He intends to sign up as a paid volunteer for a clinical trial in Sydney to finance the rest of his travels. Hannah wonders if she should advise against doing this. Having worked for years on issues of pharmaceutical safety, I ask for details during our dinner-table conversation. What kind of drug is involved? Is it an early trial? My daughter, who grew up with Fedde, joins the discussion, asking: “Why are you so concerned about him participating in a hospital experiment? Surely if something goes wrong he is in good hands. Do you know what he experiments with when he is in Amsterdam?”

I came of age in the 1970 and 1980s, and was engaged in activism against pharmaceutical company misinformation and greed. I scrutinize the safety profiles of pharmaceuticals; I buy organic food, checking its origins; and I take detours when cycling to work to avoid streets that have bad air quality. How do youth today experience the chemical environments in which they grow up? What do chemicals do for them? After more dinner-table discussions with my daughter and her friends, and exploratory talks with other young people, I became intrigued by how many young people are embracing all kinds of substances, whether to feel good, be creative, have more focus, or stamina, or meet some other purpose. How do young people “do” chemicals? And why do they do what they do?

Social and behavioral scientists have tended to focus on young people’s use of (both legal and illegal) psychoactive and addictive substances, largely ignoring their use of other kinds of chemicals. There are four broad trends in this body of research: one set of studies defines substance use as risky behavior, something that needs to be prevented by understanding the determinants of use. These studies usually have a narrow focus on particular drugs, such as alcohol, cigarettes, cannabis, heroin, and ecstasy; they present statistics on the use of these “problem drugs” and seek to identify associated risk factors. These surveys show that factors such as education, poverty, violence, and peer pressure are associated with drug use (for example, Assanangkornchai et al. 2007; Kokkevi et al. 2007; Hibell et al. 2009; Legleye et al. 2011). For example, a study of cannabis experimentation among French teenagers found that one out of five 8th to 10th graders had tried cannabis (Jovic et al. 2014). The researchers report that teenagers of low socioeconomic status who were living with both parents, feeling well monitored, and had good communication with their mothers were less likely to experiment with cannabis than those who did not like school and felt undervalued by teachers. In the Netherlands, a study found that parents had little influence on young adults’ cannabis use, which was instead associated with the actions of their peers and partners (Liebregts et al. 2013).

A second group of studies maintains that drug use is often a survival strategy for young people growing up in risky environments and under conditions of structural violence (Bourgois 1998; Rhodes 2002; Pilkington 2007; Singer 2008). For example, in urban poor communities in Makassar, Indonesia, young men consume locally brewed spirits, along with psychoactive prescription drugs, cannabis, and heroin to project “rewa”—masculine bravery. As one informant put it: “To survive and to be respected, you need to be rewa. Indeed, you’re not a real lorong guy if you don’t put on a brave face against dangers” (Nasir and Rosenthal 2009, p. 240). Lasco examines how a group of young men, working stand-by as tambays and hoping to pick up odd jobs in a harbor in the Philippines, use methamphetamine, locally known as shabu, for strength, confidence, and disinhibition. “We are not educated and we have nothing. Where will we gets the confidence to talk to others, if not from shabu?” they explain (Lasco 2014, p. 85). To help them manage irregular working hours, they use methamphetamines to stay awake, and cannabis and alcohol to fall asleep.

A third, very different set of studies examines substance use as a kind of self-optimization, fueled by pharma campaigns and neoliberal policies that call on citizens to take responsibility for their own well-being (Rose 2007). These studies are interested in how today’s youth use “brain chemicals,” to manage their feelings (Dumit 2010; Jenkins 2011). Many such studies have examined students’ off-label use of prescription drugs to manage attention deficit hyperactivity disorder (ADHD) and to reduce fatigue and improve cognitive functions. For example, fraternity members at a college in the southeastern United States use the ADHD drug Adderall off-label when they have to perform academically even though they have no medical diagnosis, as well as for recreational purposes (DeSantis et al. 2010).

A final group of studies emphasizes the pleasures of taking drugs (Moore and Miles 2004; Hunt et al. 2007; Duff 2008; Martinic and Measham 2008; Shapiro and Kirksey 2017; Bengtsson and Ravn 2018) and the role of social environments in enabling pleasurable substance use (Duff 2011; Keane 2011). For example, weekend raves allow young people to have a break from pressures in their lives (Moore and Miles 2004; Riley et al. 2010). Young people try out new “ designer drugs” to experience an intense form of pleasure, especially if users are able to avoid negative effects (Quintero and Nichter 2011; Hunt et al. 2007). Among such studies, Race’s (2009) study of ecstasy at queer dance parties has been influential; he argues that it generated pleasure, euphoria, caring, and togetherness, facilitating a form of bonding that enabled gay communities to confront homophobia and the challenges of the HIV epidemic.

## ChemicalYouth

The project that this book is about, ChemicalYouth, made a case for examining a broader range of chemicals that young people use in their everyday lives. It focuses not just on psychoactive substances—the use of which is viewed with concern by parents, educators, and policymakers—but all other chemicals that young people use to boost pleasure, mood, vitality, appearance, and health, more general dynamics that have received far less scholarly attention. It approaches the use of chemicals as situated practices that are embedded in social relations and that generate shared understandings of efficacy. More specifically, it seeks to answer the question: how do young people balance the benefits and harms of chemicals in the quest for a good life?

In our inquiries into young people’s chemical practices, we have aligned ourselves with social analyses that approach social life as ‘doing’ rather than being (Nicolini 2013). Following Schatzki (2001), we define chemical practices as “embodied, materially mediated arrays of human activity centrally organized around shared practical understandings” (p. 2). This analytical approach encompasses the wide-ranging multitude of chemical practices, and the diverse situations and concerns that fuel these practices in everyday life. Focusing on practices, we find out what young people do with chemicals and why. Our approach overcomes the limitations of the youth drug-use studies that we reviewed above, which focus on the role risk environments in drug use and on particular kinds of reasons for taking drugs (survival, self-optimization, pleasure).

Approaching chemical practices as embodied arrays of human activity further provides insights into how chemical effects are experienced and the shared understandings that emerge through the exchange of experiences and practices.^1^ We thus uncover the diverse pragmatic reasons why people use chemicals,^2^ and the trajectories through which some chemicals become “routinized way of engaging with the world,” reproduced through “repeated performances of everyday actions” (Bengtsson and Ravn 2018, p. 41). We show how over time, chemical experimentation can develop into more habitual and individualized use of chemicals, as Mimi Nichter (2015) also observes in her ethnography of smoking on a college campus. Young people may start smoking cigarettes together, but they often end up also smoking on their own to relax after a hard day’s work. When chemical use is routine, concerns about balancing safety and harm become less prominent. Users find their “ hiyang,” a Filipino term used to indicate that a user and a product are compatible (Hardon 1992).

These fine-grained ethnographies of situated chemical practices, provide profound understandings of the everyday dynamics through which youth mitigate chemical harm and navigate risk and uncertainty across diverse social worlds, contributing to a growing body of critical studies that examine the complex webs of social practices, power dynamics, gender and race relations, and inequalities that shape risk (Zinn and Olofsson 2019; Nygren and Olofsson 2014).

The ChemicalYouth project examined chemical practices in great detail. We observed how young people chewed, snorted, injected, and ingested diverse chemicals; how they applied them to their skin; and how they mixed and carefully dosed drugs to optimize effects. We asked why chemicals were used in specific ways, which led to insights into the diverse aspirations that youth try to achieve by doing chemicals as well as the conditions of precarity that fuel their use. We also probed into how they sought to prevent harm, which led to insights into how they adjusted dosages, mixed substances, and sought substitutes to balance benefits and harms.

We found that benefits and harms are not only physical. Youth also seek social efficacies, such as having the confidence to connect with clients, while seeking to avoid negative social effects, such as being stigmatized by peers for not moderating their intake of drugs. Such balancing acts are rarely individual. Rather, as demonstrated by the ethnographies undertaken as part of the ChemicalYouth project, young people try out and tinker with chemicals together, a process we have labeled “ collaborative experimentation” (Hardon and Idrus 2014). The collaborative nature of chemical use may come as a surprise, as young people might be expected to compete with each other for educational opportunities and jobs. But fieldwork suggests that desires for social bonding are stronger than competition, driving chemical use.

In the ChemicalYouth project, we examine young people’s chemical practices as collaborative experiments that involve strategies to try out new chemicals, enhance efficacies and mitigate both bodily and social harms, and to share understandings and experiences. A key insight is that youths’ collaborative experimentation with chemicals involved connecting to people across generational, professional, and spatial divides to learn about the effects of chemicals. Our observations resonate with the analysis of Callon and colleagues (2001), who point out that in an uncertain world characterized by rapid technological change, the division between professional and laypeople is outdated. They emphasize the importance of collective experimentation and learning in hybrid forums, in which professionals, experts, and ordinary citizens come together, to discuss the risks of GMO, mobile phones, and asbestos. “Everyone contributes information and knowledge that enrich the discussion” (Callon et al. 2001, p. 9). Our interlocutors consulted and contributed to online forums and websites, scrutinized package inserts and prescription guides, and sought advice from relatives or friends with medical knowledge or pharmacy backgrounds. All this work made us realize that the label “layperson”, indeed, does not properly characterize our interlocutors.

The collaborative nature of experiments is highly visible in online drug forums (Berning and Hardon 2016), as illustrated by this exchange, in which a drug user who calls himself GTCharged asks for advice on how to use Soma (a potent painkiller) on the website Drugsandbooze.com, which is dedicated to reduce harm through informed use.

*GTCharged [1 May 2010 at 20:50]*: I just got prescribed Soma250 mg. It’s a muscle relaxant. Can you snort this pill? Will it kick in faster? …
*Robert Poop [answers within 30* *minutes]*: I think it burns like a mother fucker if I remember correctly so if you can deal with the pain give it a shot. I personally never felt the need to snort it, just eating them worked great.

We examine how knowledge is generated through such forums, forming the basis of more systematic forms of “evidence” generation on the effects of new designer drugs (see Chapter 10.1007/978-3-030-57081-1_8).

## Doing Chemicals

In our analysis of how young people do chemicals, and what chemicals do for youth, we took as a point of departure the idea that efficacies are not fixed but fluid (Hardon and Sanabria 2017). Young people through their situated practices and collaborative experiments make chemicals act in specific ways. We began with the premise that chemicals are rendered efficacious in laboratories, manufacturing plants, therapeutic settings where they are prescribed, drug stores where they are sold, and everyday lives where they are used. Medical and toxicological research, commercial interests, and societal concerns all shape the effects that are actualized. Efficacies are thus made and remade in drugs’ trajectories from production to use, where young people appropriate them in their quests for the good life. The ChemicalYouth project asks how, at the end of this trajectory, youth generate new understandings of what chemicals can do, by trying them out and tinkering with them to generate specific efficacies that matter in their everyday lives. Many of our interlocutors also sold products in stores or to their peers, or worked as distributors in multilevel marketing schemes. When selling chemicals, they testified to their beneficial effects and tailored products to their clients’ needs. Social media influencers, in particular, promoted products and were compensated for their efforts by advertising agencies and manufacturers, thereby amplifying the circulation of positive information on products.

**Fig. 1.1:**
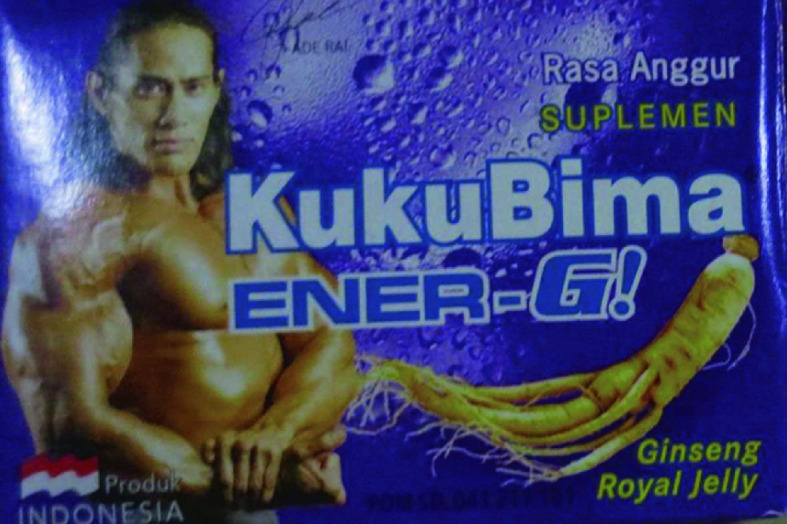
KukuBima Ener-G drink (Picture taken by Anita Hardon, October 15, 2019, Indonesia)

In examining the ever-emergent nature of chemical efficacy, we pay attention not only to how chemicals are made but also how they are made meaningful. In developing this analytical framework, we are inspired by Ingold (2012) who proposes a shift from studying objects to knowing materials, which requires following matter as it flows from one situation to the next. The chemicals used by our interlocutors are derived from plants, mined, or engineered in laboratories and production plants. Manufacturers not only mix them with adjuvants to create attractive taste, color, and texture, and to enable ingestion, inhalation, injection, or application to the skin, but they also “inform” them through labels that declare contents, benefits, and harms, and through advertisements that link products to positive effects and desires. We borrow this idea from Barry (2005), who argues that chemistry is a science of associations in which molecules are “informed.” When young people appropriate chemicals in their everyday lives, to achieve their aspirations, they also inform chemicals by producing shared knowledge on what chemicals can do and how best to use them. This shared knowledge contributes to fluid efficacies by arousing expectations of beneficial effects. Consider the packaging of a popular Indonesian energy drink called KukuBima (Fig. 1.1).

The manufacturers package the product as a sachet, containing a very sweet, grape-flavored powder and a combination of active ingredients, including caffeine and ginseng. As we elaborate in Chapter 10.1007/978-3-030-57081-1_6, some men working in the port of Makassar use this product to keep up their physical stamina, while others take it after work to enhance their virility (ginseng is known to be a potency-enhancing herbal medicine). Our interlocutors mixed the powder into water (the cheapest option) and different kinds of drinks. When dissolved in Sprite, they expected the product to kick in quickly; dissolving it in milk was thought to add nutritional content to the drink, a benefit if one suffers from fatigue. Printing virile images on packages, highlighting chemicals that are included in the sachets, and mixing the powders with milk and Sprite to achieve specific effects—all of these can be understood as “informing” the chemicals contained in the sachets.

There is a substantial body of critical social science literature that analyzes how pharmaceutical companies reinform their products to increase the market for their products. Companies link their drugs to the lifestyle desires of new groups of users, rebranding pills and attractively packaging them (Droney 2016; Ecks and Basu 2009; Quintero and Nichter 2011; Wolf-Meyer 2014). Greenslit (2005), for example, describes how Pfizer successfully reinformed its blockbuster drug fluoxetine (the active ingredient of Prozac) for the treatment for premenstrual dysphoric disorder, giving the pill a pink color and calling it Sarafem. Similarly, Hartley (2006) describes how the “pinking” of Viagra expanded the market of this potency drug to female sexual dysfunction. Martin (2006) emphasizes how informing consists of “carefully engineered images and concepts with sparse language designed to capture desires and hopes, while transposing in minuscule font the potential side effects that are not really meant to be read” (p. 282).

Critical social scientists point out that research on pharmaceutical efficacy is generally funded by pharmaceutical corporations and investment banks, which seek to increase sales and maximize profits (Dumit 2012; Healy 2006; Sismondo 2010; Sunder Rajan 2017). This scientific “informing” amplifies positive pharmaceutical potencies, while downplaying risks (Applbaum 2009; McGoey 2012). The ChemicalYouth ethnographies suggest that something similar is going on in the informing of chemicals through collaborative experiments by youth. Positive effects are amplified through face-to-face and online exchanges of experiences, sometimes paid for by manufacturers who see the potential of social media to market their products.

## Collaborative Inquiry

Throughout this book, I honor the intensely collaborative inquiry that has characterized this project by referring extensively to our published studies and using the pronoun “we” to refer to our work and our insights. Each chapter provides a box featuring the short biographies of the youth ethnographers whose case studies are highlighted in the chapters. The full studies are available at chemicalyouth.org, the project’s website, and we encourage readers to consult it for more specific information.

The research team conducted multisited fieldwork in Amsterdam, Paris, Makassar, Yogyakarta, Cagayan de Oro, Puerto Princesa, and Brooklyn from early 2013 to late 2018, with additional ethnographies conducted by an associated researcher in Addis Ababa. These eight urban centers are magnets for young people who seek to study, work, and make their future; they are “innovative, unchartered borderlands along which the global meets the local” (Comaroff and Comaroff 2000, p. 8). They are spaces of interaction that gather young people, ideas, and material objects and practices from around the world, creating gateways to a wider world of opportunities (Nilan and Feixa 2006; Hansen 2008). Our interlocutors faced diverse challenges and led multifaceted lives—as students, workers, designers, lovers, and social media influencers—that changed over time, along with their educational trajectories, work engagements, migration processes, and social affiliations. They connected to each other and to opportunities through kinship networks and social media, facilitated by mobile phones and their rapidly increasing access to the internet.

A key premise of the project was that, by examining how and why youth “do” chemicals, we could gain insight into the socialities that make up their lives and their shared aspirations for a better future, while also learning about the challenges and vulnerabilities they experience. Following Butler (2004a), we use precariousness to refer to vulnerabilities that emerge from life itself, in the sense that we need others to survive. While precariousness is shared, it is not the same for all. Young people’s control over their destinies is affected by poverty, unequal access to education and health systems, the globalization of capitalist production, the rise of information technologies, the flexible labor arrangements that emerge along with these trends, and gender-, sexuality-, and race-based discrimination, which, taken together, are also referred to as “ precarity” (Lorey 2015).^3^ Precariousness thus refers to the condition of vulnerability shared by youth, while precarity refers to the regulatory, labor, welfare, education and health structures that shape these vulnerabilities (Vallas and Prener 2012; Han 2018).

We examined across our sites, the regulatory structures that do or do not protect young people from chemical harm, gender dynamics and racial inequalities that fuel consumption of hazardous products, and labor policies, all of which render their lives insecure. The differential precariousness of our interlocutors’ lives has become painfully clear in the COVID-19 pandemic, which is raging while I am finalizing this manuscript. Across our sites young people are losing their jobs, with huge variations in their capacity to protect themselves and each other from the new coronavirus, and large inequalities in opportunities for government support to mitigate the economic downturn.

Across the urban centers where we did fieldwork, youth encountered a bewildering array of chemical products in drugstores, supermarkets, pharmacies, and online shops, which they strategically used to feel well and remain productive at work. And, they are targeted by advertisements for beauty products, pharmaceuticals, (e-)cigarettes, (energy) drinks, vitamins and supplements through their Instagram and Facebook accounts, TV and radio, and by posters hanging at neighborhood stores. The images that circulate through these media encourage “imagination and consumption” (Appadurai 1996), while at the same time sharpening feelings of exclusion and marginality among those who have not achieved their aspirations and cannot afford the chemical goods (Comaroff and Comaroff 2000; Cole and Durham 2007). The advertising images moreover amplify the potential benefits of chemicals, rendering any toxicities they may entail invisible.

Our interlocutors were roughly between the ages of 18 and 30 when we conducted fieldwork. Born in the late 1980s and 1990s, they grew up in a period when new communication technologies and the gig economy dramatically changed young people’s lives throughout the world. Their futures were connected through global markets, exploitative labor arrangements, and “flows of signifiers and practices that make up contemporary youth cultures” (Farrugia 2018, p. 3). Many conducted outsourced labor and work 24/7 through internet technologies and new labor platforms. Their situated chemical practices, movements across literal and imagined spaces, and connections to global markets and commodity chains joined them to global “youthscapes” (Maira and Soep 2004).^4^

## Navigating Precarity

Although increased access to education, and images that they view online fuel dreams for a better future, the precariousness of their everyday lives led the youth we spoke with to doubt if their aspirations could really be achieved (see also Butler 2004b; Vallas and Prener 2012; Lorey 2015). Many young people inhabit a commercially mediated “nowhere place” between a devalued local past and an unreachable future (Liechty 2003). The future becomes even more bleak in settings where people are directly affected by the impact of climate change, including Cagayan de Oro and Makassar, where heavy floods have resulted in increased economic insecurity for young people and their kin.

Across the urban sites where we conducted fieldwork, young people have responded to these challenges by seeing themselves as “flexible collection of assets,” which they hoped to develop to “position themselves in a rapidly shifting global economy” (Martin 2000, p. 582; see also Gershon 2017; Urciuoli 2008). To increase their chances of success, they were eager to learn new competencies, make new local and global connections, refashion their styles, and groom their faces and bodies (Liechty 2003; Rofel 2007; Lukose 2009; Cole 2010; Newell 2012; Hann 2018). Aware that the world was changing rapidly, they were willing to “sacrifice, work, invent and negotiate for a future different, and better than the one they live in now” (Durham 2008, p. 947). These desires for a better future made them an easy target market for corporations seeking to sell a whole range of chemicals, deploying advertisements to reinforce and fuel desires through an ever-expanding range of communication channels. At the same time their eagerness to succeed in life spurred them to set themselves up as mediators and movers in the same commodity markets, if only to make some money to pay for their chemical needs.

While job prospects differed across the urban sites, our interlocutors shared concerns about precarious labor conditions. Most had to contend with temporary employment, as technological advances have made routine and manual jobs scarce. Across our field sites, the service sector was the largest employer of youth, providing both formal and informal jobs in banks, malls, markets, household services, the emerging wellness industry, transport, restaurants and bars, and more. In the European cities, labor laws and state unemployment benefits offered some protection, while in the Asian cities being jobless or having to tend to family ( health) crises could result in acute poverty. As a result, their educational careers and economic aspirations could be seriously disrupted.

Across our ethnographic sites, we explored the situated social and economic dynamics that contributed to youth precariousness. This precariousness concerns existential anxieties about the wellness of their bodies and minds, as well as concrete fears of “politically and economically induced precarization, fear of unemployment or not being able to pay the rent or health care bills even when employed” (Lorey 2015, p. 131). Chemicals provided our interlocutors a sense of control as they faced multiple insecurities and challenges in their everyday lives, which explained why they invested their scarce resources in such products.

We found that many of our interlocutors ended up worse off than when they started their chemical “investments.” While our interlocutors thought the products that they bought in supermarkets, pharmacies, and online markets were safe, sadly, all mechanisms over the world to prevent hazardous chemicals from being sold are weak, because the economic interests of pharmaceutical and tobacco corporations, vitamin and supplement manufacturers, and beauty product companies exert a stronghold on regulatory processes. As a result, though it was often not immediately evident, our interlocutors ran health risks due to their long-term exposure to multiple toxic substances (which may interact with each other to cause even more harm), while the benefits they gained from their chemical investments were often minimal.

Regulatory protection from chemical harm was uneven across the research sites. While governments take responsibility for the safety of some chemicals, notably pharmaceuticals and narcotic drugs, we found that beauty products, energy drinks, herbal medicines, food supplements, and vitamins are regulated much less stringently. For these products, pre-market approval is easy to get, as long as the companies avoid including certain chemicals, such as mercury, that are known to cause serious harm. In some countries, there have been regulatory moves toward adopting the “ precautionary principle,” a cautious strategy of pausing and reviewing before allowing new chemicals on the market (Read and O’Riordan 2017), but implementation of this principle has proven to be difficult for government regulators.

## Head to Toe

To ethnographically explore the expansive range of chemicals that matter to youth in their everyday lives, and gain insights in the ways in which the confront existential uncertainty and chemicals risks we started fieldwork with a new research instrument the researchers dubbed the “head-to-toe interview.”^5^ We asked respondents to take us on a “ grand tour” of every chemical they used for their hair, eyes, face, lips, teeth, bodies, ending with their toenails. The systematic treatment of the human body prevented feelings of shame or fear for repercussion when talking about chemical practices and body parts that might be more sensitive. This method helped us tune into the role that chemicals played in our interlocutors’ lives, including the social relations in which a given use was embedded, and the aspirations and challenges reflected in the practice. We asked our informants what they sought to achieve through chemical use (also probing into their more general aspirations in life), the advantages and disadvantages of different products on the market, and how they learned about and acquired them. Their responses allowed us to identify themes for further enquiry.

The second phase of the project involved focused ethnographies of specific chemical practices that emerged as central in the everyday lives of particular subgroups of youth. We collected data through interviews, participant observation, and “four-day recalls” in which our interlocutors meticulously tracked the substances they used. We also conducted feedback and validation sessions, where we discussed emergent findings with our interlocutors. Together we identified potential foci for ethnographic observation, based on the findings of the grand tours, thereby co-producing a body of knowledge, as collaborators rather than as field assistants, which is a second way the ChemicalYouth project engaged in collaborative experimentation. The project thus not only described ethnographically our interlocutors’ collaborative experiments with chemicals, but it also engaged them in gaining insights into and analyzing these situated practices. The youth ethnographers received intensive guidance during fieldwork and have published their case studies in edited volumes, special issues, and on the project’s website; most of their publications are open access (www.chemicalyouth.org).

The feedback and validation sessions were done in karaoke bars or other public spaces that allowed for some privacy. During these, we further explored our interlocutors’ engagements with chemicals; we brought samples of commonly used products (only legal ones) and asked our interlocutors to sort them into piles, listening closely as to why products were grouped together. The physical presence of the products in the group discussion signaled to our interlocutors that we knew what mattered to them. On one occasion, one of our informants exchanged a full bottle of vaginal cleansing liquid with a half-empty one from her purse, which led us to ask who else had half-empty bottles in their purses; all of the female participants did. On another occasion we were confronted with the ethically compromising interaction with a heavy drug user taking a tablet of buprenorphine (a heroin substitute) with him to the toilet, despite our entreaties to not do so. When he came back he told us that he had crushed the pill, diluted it in mineral water, and injected the fluid to treat his withdrawal symptoms. This taught us to not bring heroin replacement drugs to these group discussions. The event also led to a discussion on safe injecting practices.

The focused ethnographies described everyday situations in which these young people studied, worked, socialized, sought partners, and engaged in sexual relations. The settings included bars, street corners, nightclubs, music festivals, private homes, shopping malls, construction sites, universities, and the internet. Across the research sites, our core research questions included: When do youth use which chemical substances? How do they use, adjust, and make them? What effects do they seek and why? How do they manipulate chemical substances to modulate their effects? What adverse effects do they experience and what strategies do they use to avoid or lessen drug-related harms?

 Collaborative contrasting analysis was a key feature of the project. Anthropology has long been committed to understanding particular practices and beliefs in bounded cultural settings (see, e.g., Abu-Lughod 1991). However, understanding global phenomena, such as the widespread use of chemicals by youth, “requires approaches that can not only identify the effect of the outside on the ‘local’ but also show that the effects operate differently in various locations” (Besnier and Guinness 2020, p. 201). Documenting these differences allows us to compare “complex and diverse configurations of categories and processes” (Besnier and Guinness 2020, p. 212).

By conducting collaborative ethnographic research across various local sites, we were able to engage in an iterative analysis of similarities and differences in situated chemical practices. This process sharpened the ethnographic inquiries that were done. Together the youth ethnographers examined how phenomena can play out differently in different locales; this we refer to as “ collaborative contrasting analysis.” Why, for example, were our female Indonesian respondents just interested in whitening their faces, while in the Philippines women wanted to whiten their whole bodies? And why did Filipino men also whiten their skins, a practice most Indonesian men would frown upon (Chapter 10.1007/978-3-030-57081-1_5)?

Young people across our field sites used chemicals for three main reasons: to achieve wellness, to enhance work opportunities and capacity, and to try out different kinds gender identities and sexual ways of being in the world. Achieving wellness entailed using products to feel attractive, connected, happy, and healthy, and to experience what we refer to elsewhere as “hassle-free” highs (Hardon and Hymans 2016; van Schipstal et al. 2016; Hardon et al. forthcoming, 2020). We found that it also involved using chemicals to create and enjoy lean and muscular bodies. Trying out different kinds of gender identities and sexual ways of being in the world involved multiple ‘ chemical sexualities’ (Chapter 10.1007/978-3-030-57081-1_4), including using hormones to grow breasts and using drugs to enhance sexual experiences. In terms of enhancing work opportunities and capacities, we found that youth across the sites used chemicals to achieve what is euphemistically called a “pleasing personality” (Taqueban 2018), or in other words a physical expression that pleases clients. They used chemicals to feel confident, be creative, have focus, and enhance stamina, and in doing so they built up biocapital, the value generated in capitalist modes of production through investments in biological materials. This investment-oriented logic highlighted the precarious labor arrangements (Hewison and Kalleberg 2013) that shaped young people’s use of chemicals to enhance their productivity. In seeking wellness and productivity, our interlocutors joined in the quest for “the good life” (Gregory and Altman 2018), a pursuit that characterizes the everyday lives of so many young people who struggle to make a living in times of precariousness.

Our youth ethnographers did extensive participant observation and semi-structured interviews.^6^ All interviews were recorded and transcribed. Transcripts were stored in NVivo and analyzed in teams, with the research team reading each other’s interviews in analysis sprints, to jointly generate core themes for analysis. We exchanged notes from our field research in Asia, Europe, and the United States, and read each other’s transcripts, seeking core themes for further analysis, and held workshops to interpret the emerging insights. We asked: why are some practices similar and others different? Together we submitted contributions to special issues and edited volumes. In the publishing process, we received editorial support from Takeo David Hymans, a science writer who was involved in the ChemicalYouth project from the beginning.

**Fig. 1.2:**
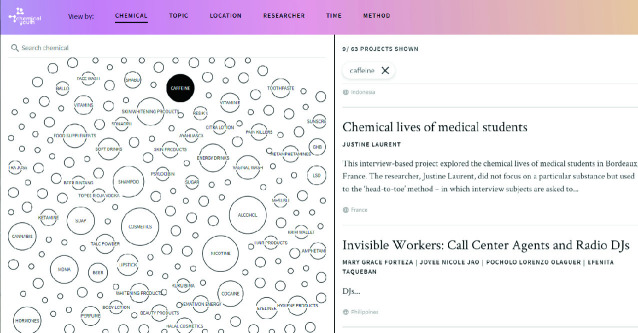
Screenshot of an overview of the chemicals that figure in the reports of the youth ethnographers. Clicking on a bubble calls up the corresponding results for the whole project (Photo taken by Anita Hardon, January 2020, the Netherlands. ChemicalYouth 2020. www.chemicalyouth.org)

Our fieldwork was multimodal by design. We engaged youth in photography projects, and we conveyed your insights through documentary films and exhibits. The findings are also available on the project’s website, organized by chemical, topic, location, researcher, and methods (Fig. 1.2).

The website displays the full range of chemicals, locations, topics, methods, and researchers that make up the ChemicalYouth project. It enabled contrasting analysis across our sites, as well as the writing of this book. It was made to inspire future researchers by providing access to the multitude of open-access publications that we made together, as well as still unpublished work, and the audio-visual arts projects that emerged from the project.

## Unevenness of Harm Protection Mechanisms

While studying the chemical lives of young people, we were struck by the unevenness of the regulatory strategies for different kinds of chemical products, not only between the countries where we did fieldwork, but also within countries. In all our sites, consumers were warned about the adverse effects of some substances, while receiving no cautionary information regarding others (Singer 2007; Homburg and Vapeul 2019). Why are some chemicals with exaggerated health claims allowed on the market, while the efficacy claims of others are tightly regulated? Why are some chemicals ruled illegal, while other equally hazardous ones are allowed to seep into our environments?

In the United States, where many cosmetics and food supplements are manufactured, the Food and Drug Administration (FDA) maintains that manufacturers “have a legal responsibility to ensure the safety of their products” (U.S. FDA 2005). All companies are required to do is list the ingredients on packages; they do not need to submit proof of safety to the government. The European Union has adopted the precautionary principle in its Registration, Evaluation, and Authorization of Chemicals (REACH) Policy, which requires manufacturers to provide evidence of a product’s safety before it is allowed on the market, and doing so has led to the banning of 1328 chemicals from cosmetics that are known or suspected to cause cancer, genetic mutation, reproductive harm, or birth defects (Homburg and Vapeul 2019). In contrast, the US FDA has only banned or restricted 11 chemicals from cosmetics (Campaign on Safe Cosmetics 2020). In the Asian region, the cosmetic directive (adopted in 2008) regulates the use of only three chemicals: mercury, lead, and arsenic (Milman 2019).

Manufacturers have been successful in preventing the adoption of the precautionary principle in the United States (MacKendrick 2018), despite the 1976 adoption of the Toxic Substances Control Act (TSCA), which assigned the Environmental Protection Agency (EPA) the responsibility to control commercial and industrial chemicals that pose “unreasonable risks of injury to health or the environment,” by 2005 the agency had only restricted the use of five chemicals (United States Environmental Protection Agency 2016). The TSCA was toothless from the moment it came into force. It oversees over sixty-two thousand chemicals that were in use prior to the bill being signed into law. Once chemicals are on the market, it is very hard for governments to restrict their use, as they then have to prove the product’s adverse effects on human health or the environment.

Narcotic drugs, namely drugs that are potent psychoactive substances, are the most strictly regulated and most aggressively informed substances in all of the countries where the ChemicalYouth project was conducted, which paradoxically gave our interlocutors the impression that legally marketed cosmetics, energy drinks, and supplements are safe. Each country has a list of scheduled drugs. Drugs such as cocaine, heroin, methamphetamine, ecstasy, cannabis, and LSD are often included on such lists. Governments ban these drugs because they are seen to have no medical utility and because they can cause addiction and/or cause serious adverse effects. But the lists change, as regulatory agencies reassess evidence and in response to contestations.

**Fig. 1.3:**
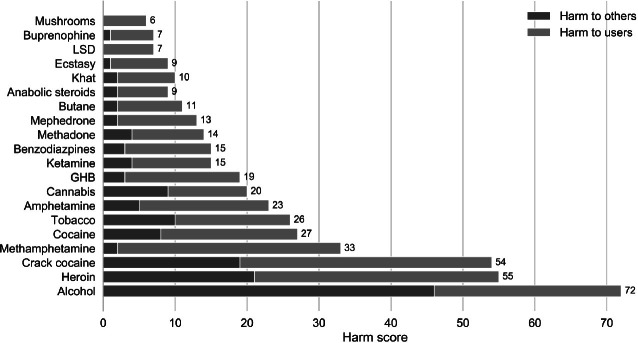
Graph reproduced by Javier Garcia-Bernardo with original data from Nutt and colleagues (2010)

The Independent Scientific Committee on Drugs (ISCD), based in the United Kingdom, scored 20 psychoactive drugs on 16 criteria, nine related to harms to individuals and seven concerning harms related to others. Both categories of harm included physical, psychological, and social dimensions. The experts concluded that the drugs most harmful to individuals were heroin, crack cocaine, and methamphetamine, whereas the drugs most harmful to others were alcohol, followed by crack cocaine and heroin (Fig. 1.3).

The interesting point in this scoring by experts is that alcohol, a legal drug, is scored as the most harmful drug, whereas cannabis, ecstasy, and LSD have relatively low scores for harm to users and others.

 Indonesia and the Philippines have declared a War on Drugs, and both users and dealers have been sentenced to the death penalty. In contrast, France and the Netherlands prosecute drug users in less severe ways. The Netherlands stands out with a tolerant drug policy that distinguishes between hard drugs like heroin and ecstasy, which are entirely prohibited, and soft drugs like cannabis, the sales and use of which are tolerated (though commercial production is illegal). Many countries (including Uruguay, Canada, and several states in the United States) have recently taken cannabis off the list of scheduled drugs, based on evidence that it can be used safely. As a result, cannabis products are now being re-classified in many different ways (Caulkins and Kilborn 2019).

**Fig. 1.4:**
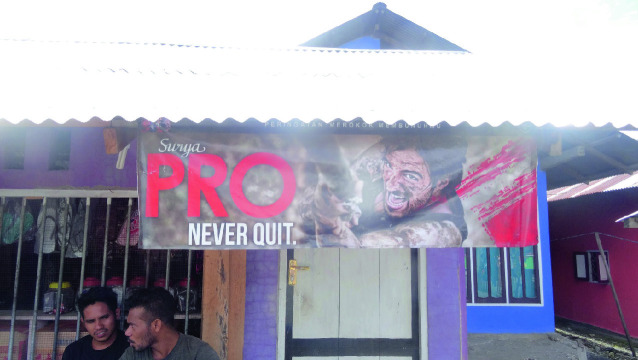
Banner for Surya Pro, which suggests that brave men never quit (Photo taken by Anita Hardon, December 30, 2018, Morotai, Indonesia)

 Tobacco is also a heavily regulated substance. The global consensus on nicotine’s addictive properties and the severe adverse health outcomes associated with (secondary) smoking have led to a global treaty on tobacco control, called the World Health Organization (WHO) Framework Convention on Tobacco Control, which came into effect in 2005. The United Nation’s Sustainable Development Goals, adopted in 2015, call on governments to strengthen the implementation of the treaty in all countries, a target that is monitored by measuring the prevalence of current tobacco use among people aged 15 years and older (United Nations 2015). The Philippines, the Netherlands, and France are adopting the recommendations of this treaty, including those that aim to protect youth from tobacco advertising. However, Indonesia (a country with a large tobacco industry) has not signed the treaty, and in that country youth are heavily targeted with advertising for cigarettes (Fig. 1.4). In all countries, e-cigarettes are increasingly used by young people as a substitute for cigarettes, partially due to advertising that is not constrained by the global tobacco treaty.

If chemicals make medical claims, as is the case with pharmaceuticals, governments demand strong evidence of safety and efficacy before allowing them on the market. The evidence has to involve at least two large-scale clinical trials. The safety-efficacy profile also determines whether a pharmaceutical can be sold over the counter or only by prescription. If a drug is categorized as a prescription drug, it may not be advertised directly to consumers (except in the United States). But the enforcement of these regulations differs wildly. In Indonesia most prescription drugs can be bought over the counter, which is how the young people we conducted ethnography with gained access to a wide range of psychoactive prescription drugs.

Despite the pre-market approval process, adverse effects often only become visible when the products are used in larger populations, and among groups of people who were not included in the clinical trials. In an ideal world, post-market surveillance would pick up these adverse effects, but such studies are rarely conducted in our Asian field sites, and information on adverse effects that are occasionally reported by users and observed by doctors are generally not widely publicized, because drug surveillance systems are not in place or not functioning well.

A pharmaceutical’s regulatory status is revisited when adverse effects are reported, and when post-marketing surveillance provides evidence of harm (Abraham and Lewis 2000; Davis and Abraham 2013). Food and drug authorities have standing committees that review new evidence and decide if the regulatory status of a product needs to be revised. One reason for revision can be a drug’s potential for abuse. For example, the potent painkiller carisprodol came on the market more than 50 years ago as a pain medication for the treatment of lower back pain and other indications. However, US doctors increasingly reported withdrawal symptoms—including vomiting, anxiety, insomnia, and hallucinations—among patients who discontinued carisoprodol (Substance Abuse and Mental Health Data Archive 2003; DEA 2011). In Europe, a study by Bramness and colleagues (2007) found that carisoprodol was hugely overprescribed and that patients often received their prescriptions from multiple doctors. The drug was subsequently taken off the market in Sweden (2007) and Norway (2008), and the European Medicines Agency recommended that member states stop authorizing carisoprodol for the treatment of acute back pain (Hardon and Ihsan 2014). But often, companies contest the regulatory proposals, making it difficult for regulatory agencies to take action. As this book shows, such an uneven regulatory landscape means that drugs prohibited in some places still circulate widely; indeed, carisoprodol, under the brand name Somadril, was one of the more common drugs taken among the youth we studied in Indonesia.

The ChemicalYouth ethnographies drawn upon in this book reveal that young people use a wide range of chemicals to enhance wellness and enable work, and that they develop elaborate techniques to increase benefits and avoid harms. They do so in an ecosystem that informs different categories of chemicals in very different ways. For some chemicals, warnings about harms are amplified by drug authorities and health educators, while for other kinds of chemicals, such as beauty products and supplements, benefits are overly touted and risks dangerously downplayed.

## What Follows

In each of the following chapters, we draw on our collective ethnographic research to show how young people ‘do’ particular kinds of chemicals and why they do so. Each chapter contrasts ethnographic insights from at least two different countries and multiple focused ethnographies. Our method of following chemicals from one country to another provided insights into the site-specific conditions of everyday life and regulations that shape use. We learned how chemicals are ingested, inhaled, and injected, and how they are also mixed to enhance effects. Across the sites, we examined how shared understandings of efficacy emerged and how knowledge produced in scientific studies intersected with these more popular understandings. We scrutinized safety and efficacy claims made in advertising campaigns and on product packaging. By doing so, we saw firsthand how the amplification of benefits in advertising and social media contributed to the widespread use of more ordinary chemicals, such as food supplements and e-cigarettes, and how the lax regulation of such products amplifies the already existing precarization of young people’s lives.

The analysis of young people situated chemical practices presented in this book is located at the intersections of youth studies, anthropology and science and technology studies, disciplines from which we borrow concepts and offer new understandings. More specifically, this book offers an ethnographic contribution to the critical studies of risk and uncertainty (Zinn and Olofsson 2019). Starting from in-depth inquiries into the precariousness of young people’s lives and understandings of how chemicals are used tools in their quests for a good life, we examine how youth navigate chemical harms in a social world structured by inequalities and social forces which often keep them in the dark about the toxicities that they face. We contribute to the critical study of risk and uncertainty by ethnographically examining the situated chemical practices of youth, and by building an analytical framework for understanding the risks that they face and the harms that they mitigate, from in-depth understandings of their engagements with chemicals in their everyday lives.

The first empirical chapter in this book, entitled “Chemical Highs,” zooms in on the situated practices and techniques that enable some youth to enjoy the positive effects of narcotic drugs while avoiding their adverse events. In Amsterdam, for example, youth invented and employed distinct techniques in their quest for hassle-free highs. These included innovative and precise methods for dosing and administering drugs. Our interlocutors trusted their peers and had confidence in their collective techniques to determine the quality of substances. If they experienced adverse effects, they had confidence that their friends, coworkers, and online acquaintances would watch over them until the effects waned. And we also looked at how trust could be increased between youth and authorities: in Amsterdam, city authorities work with young people to carefully design harm reduction programs, which earn the participation of partying youth.

We contrast the quest for hassle-free highs in Amsterdam with the situation in Indonesia, where a severe drug war is being waged by the government that involves a different kind of risk. In Indonesia, young people commonly use psychoactive prescription drugs to get high, and they consider these safer than illicit drugs, because those can lead to the death penalty. Like their peers in Amsterdam, they seek hassle-free highs, but they lack information to protect themselves against harm—many have become addicted to psychoactive prescription drugs, without knowing that this could happen—and they don’t have a trusted partner in government.

Chapter 10.1007/978-3-030-57081-1_3, “Chemical Breath,” contrasts the smoking practices of young cannabis users in Makassar with those of young people who have turned to e-cigarettes in Paris. In both cases our interlocutors valued the bonding that happens in this shared practice and the gustatory pleasure of inhaling from joints. But in Makassar, the students who consumed cannabis were worried about the risk of attracting police attention and being imprisoned. We observed the introduction of synthetic cannabis-like designer drugs in Indonesia, ordered online, that are mixed into branded cigarettes and smoked with less risk of criminal prosecution, but more severe harm to their health. In France, e-cigarettes are increasingly popular among young smokers who want to avoid tobacco-induced harms, while continuing to enjoy the social relations that are fostered by smoking together.

In both cases, social media amplified the positive effects of the products, while information about their potential harm did not circulate as freely, thus contributing to the precarization of young people’s lives. Both cases also point to the need for sensible government regulation. Indonesia’s drug policy, which criminalizes cannabis, caused youth to take severe health risks, while the lax regulation of e-cigarettes similarly put youth at risk in Europe.

In Chapter 10.1007/978-3-030-57081-1_4, “ Chemical Sexualities,” we turn to the chemicals used by young people to mold their sexual being in the world. With ethnographic vignettes from six focused ethnographies conducted in the Philippines, Indonesia, Ethiopia and France, we show how chemicals are used to try out sexual identities, enhance sexual experiences, and prevent unwanted pregnancies and sexually transmitted infections. Young women used substances to tighten and cleanse their vaginas; men sought to “last longer” by turning to penile wraps and tissues, and a plethora of virility-enhancing drugs. In Paris, we observed how young gay men engaged in “ chem sex,” which involves injecting drugs during (often unprotected) sexual encounters. Our interlocutors explained how the practice of injecting drugs was entangled with their search for love. Feelings of euphoria and “love fusion,” common when new relationships blossom, often encourage risk taking within couples, where syringe sharing is experienced as a sign of trust (Amaro 2016). Looking through the lens of collaborative experimentation, we see how young people observed effects in their own bodies and then shared their lived experiences. We suggest that sexual health programs should acknowledge the sexual desires and health needs that are reflected in such chemical practices, and develop more chemical products to meet these needs.

Chapter 10.1007/978-3-030-57081-1_5, “Chemical Whitening,” looks at skin-lightening practices, which we found to be the most prevalent among service-sector workers in the Philippines and Indonesia. Many of our interlocutors explained this practice as a way trying to “please” their clients and employers. By having light skin, service-sector workers aimed to portray the good life that they were selling to their clients, while also performing the “pleasing personality” that their companies expected. Our analyses revealed skin whitening to be an elaborate and expensive process, involving layers of whitening via soaps, scrubs, and lotions. However, the practice differed across sites in the Philippines. While only women used to whiten their skin, more and more young men have begun engaging in this practice. Many do so because they are competing with women for service-sector jobs. Some are also inspired by the androgynous masculinities that have begun circulating in the Philippines, influenced by Korean popular culture. Seeking a lighter skin involves economic costs and has adverse effects as potent and cheap products bought on the black market, often containing banned products such as mercury. The chapter argues that while skin lighteners may be used to increase one’s value in the service-sector economy, their use can lead to the further precarization of young people’s lives. The chapter ends with a description of initiatives that seek to counter the colorist marketing of skin-whitening products and celebrate skin diversity.

Chapter 10.1007/978-3-030-57081-1_6, “Chemical 24/7,” presents the chemical lives of night workers—producers, promoters, DJs, hosts, artists, performers, drag queens, musicians, stage managers, bartenders, hospitality girls, and dancers—in Amsterdam (Netherlands), Brooklyn (United States), Bira ( Indonesia), and Puerto Princesa (Philippines). In Brooklyn and Amsterdam, young people’s work entailed producing the social spaces where partygoers can enjoy themselves. Our interlocutors used stimulants to stay alert at night, to be friendly to customers regardless of their mood, and to engage with audiences when performing on stage. In the karaoke bars of Puerto Princesa and Bira, hospitality girls and dancers were required to consume “ladies’ drinks” along with their customers. Our interlocutors employed tactics to prevent becoming drunk, including the covert sharing of drinks and teaming up with barmen to dilute their drinks with water. This chapter also examines the work conditions that perpetuated this chemical use, and the precariousness caused by night work and heavy caffeine use, which can lead to serious health conditions. It ends with a call for occupational health programs to acknowledge the 24/7 demands on workers, to seek to prevent the overuse of stimulants, and prohibit unethical ways of selling beer.

In Chapter 10.1007/978-3-030-57081-1_7, “Chemical Supplements,” we examine how the demands of service-sector labor, the strain of night work, the excitement of weekend raves, and growing concerns about environmental toxins all result in a sense of vulnerability among youth, fueling a felt need for supplements. Aggressively marketing to young people through online commercials, mass media, and street-level vitamin stores, marketers capitalize on these fears. Youths across our field sites took vitamin C to prevent colds and coughs, and to generate energy; vitamin E to gain radiant skin; and multivitamin capsules for shiny hair. Vitamin-fortified energy drinks are especially popular among construction workers and porters, both engaged in heavy physical work. Our respondent consume more complex food supplements to increase muscle mass, such as fruit shakes that were combined with ginger extract, turmeric, and honey to strengthen immunity. Young people worried about the lack of nutrients in their fast food noodles, burgers, and pizzas, and supplementing was thus a rational strategy when there was rarely time to cook. Two contrasting ethnographic vignettes—of youth who sold supplements in a multilevel marketing sales pyramid in Puerto Princesa and a young woman in Amsterdam who sold vitamins and protein powders to female body builders—show how young people co-created products to alleviate this perceived vulnerability, how they tailored products to their clients’ needs in face-to-face and online interactions, and how they consumed the products that they sold and created in order to be able to personally testify to their effects. This chapter reflects on the economic costs of supplement use in young people’s lives, and the exploitative labor arrangements that drew youth to participate in multilevel marketing .

In Chapter 10.1007/978-3-030-57081-1_8, “Chemical Creativities,” we discuss how, using novel virtual ethnography techniques, the ChemicalYouth project examined popular online drug forums where users, mostly men, share their experiments with chemicals. In this chapter, we zoom in on the online discussions about microdosing of LSD and psilocybin to enhance creativity, a common practice among young people in creative, academic, and tech environments. We present users’ narratives which show how they “do” microdosing, what they want to achieve by using these substances, and what they do to prevent or reduce harm. Collaborations between researchers and users aggregate users’ experiences online, which form the basis for clinical trials that compare the effects of psychoactive substances with those of placebos.

“Chemical Futures,” the concluding chapter, shows how young people mobilize to reduce the adverse effects of chemicals in their everyday lives. Here, we focus on a group of activists in France called Générations Cobayes (Guinea Pig Generations) and their campaigns on endocrine-disrupting chemicals. Building on this case, we take stock of the ways that young people across our field sites sought to mitigate chemical harm, showing how, unlike the Cobayes, they generally did not realize how toxicities can compound one another or act slowly, over time. We further describe how youth in their everyday lives sought to mitigate harm “from below,” though constrained by industrial strategies that amplify chemicals’ benefits through marketing and render risks invisible (Proctor 2011; Healy 2012). We propose the more widespread adoption of the precautionary principle before allowing chemicals on the market, and for building on and supporting young people’s collaborative experiments in harm reduction. Young people have been induced by manufacturers to believe in and promote the benefits of many chemicals, and governments have allowed these products on the market. When adverse effects become apparent, they tend to be dealt with one chemical at a time. Can academics, policymakers, and the concerned public all engage with youth to spread precautionary tales beyond those related to narcotic drugs, while attending also the combined risks of chemicals and slow toxicities to enhance the safer use of chemicals?

## International ChemicalYouth Research Coordinators

**Fig. 1.5:**
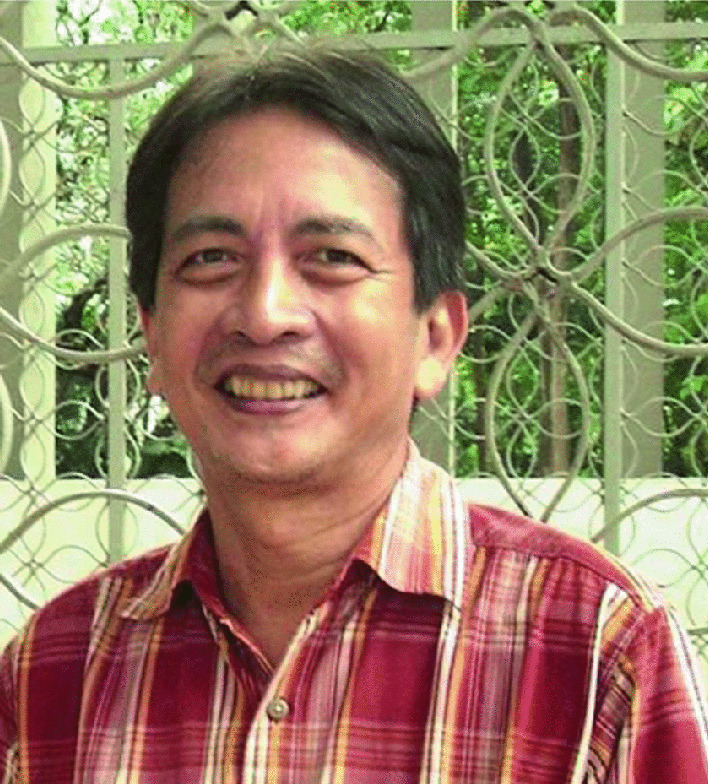
Michael Tan

Michael Tan was the Principal Investigator for the ChemicalYouth project in the Philippines. In this role, Michael conducted the analysis of skin whitening practices, supervised one ChemicalYouth PhD project, organized the site visits, and co-edited and book launch of *Making bodies work: Young people’s everyday body management in urban Mindanao* in the Philippines. He is a Professor of Anthropology at the University of the Philippines Diliman. He also served as the Chancellor from 2014 to 2020 (Fig. 1.5).

**Fig. 1.6:**
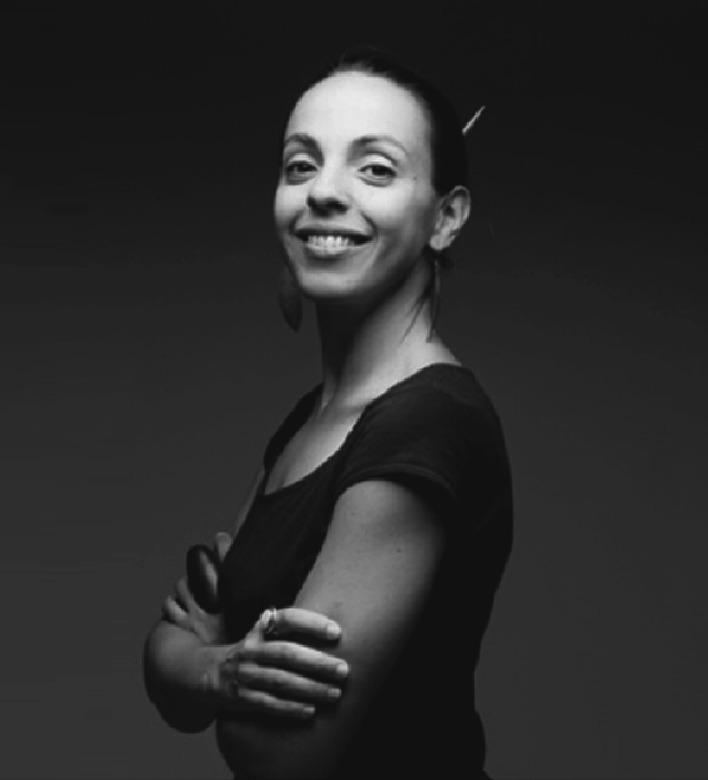
Emilia Sanabria

Emilia Sanabria was the Principal Investigator for the ChemicalYouth project in France and co-supervisor to two ChemicalYouth PhDs. She is a Senior Researcher at the Centre National de la Recherche Scientifique in Paris. She has worked at the intersections of the anthropology of health, care, and the body and science and technology studies (STS) on topics ranging from sex hormones, menstruation, and pharmaceutical cultures to obesity, nutrition, non-ordinary states of consciousness, and the psychedelic renaissance. Emilia is currently the Principal Investigator of a project on the new therapeutic uses of the Amazonian psychoactive brew ayahuasca (Fig. 1.6).

**Fig. 1.7:**
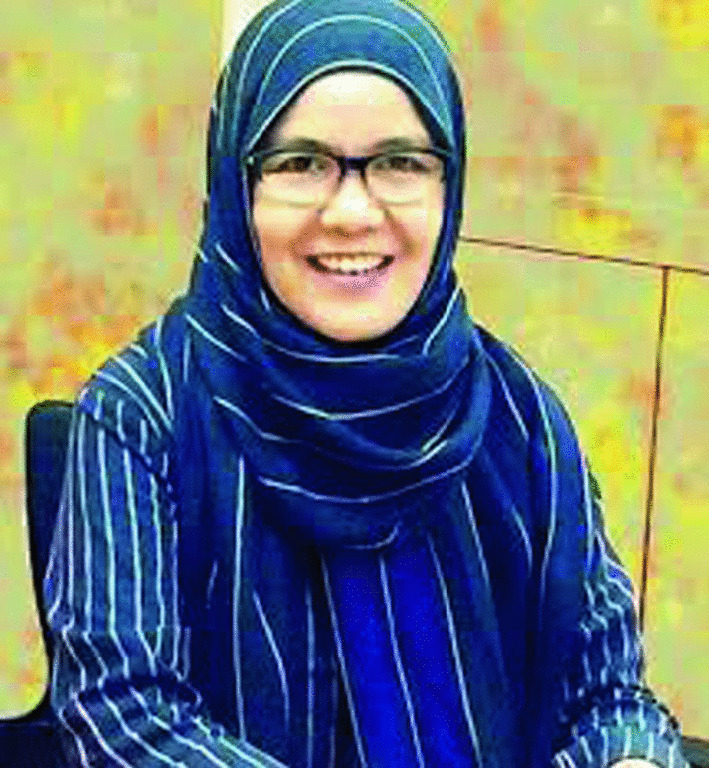
Nurul Ilmi Idrus

Nurul Ilmi Idrus was the Principal Investigator for ChemicalYouth in Indonesia and coordinated the Grand Tour there, as well as the students’ fieldwork and reporting. She is a Professor of Anthropology at the Hasanuddin University in Indonesia. Her areas of interest for research include health, gender, and sexuality. Idrus has collaborated with Anita Hardon both in the field and writing, and several of these publications are essential to this book and presented throughout (Fig. 1.7).

### Notes

1. See Nichter (2003) for an incisive analysis of risk taking, vulnerability and harm reduction among people in diverse cultural settings, who confront different kinds of health issues.
2. Thevenot (2001) points to the importance of ‘pragmatic regimes’ in every day life. He invites scholars to empirically differentiate between kinds of material engagements between agents and their environments in order to gain insights into the concept of good that underlies practice. This is an analytical strategy that inspired me in developing the ChemicalYouth project.
3. The term emerged in the 1980s in Europe to describe moves towards more flexible, globally connected labor arrangements and weakening labor protection mechanisms and welfare provisions. The term indexes a loss of labor security and stable jobs, which arguably ‘only certain countries, at certain historical periods, and certain workers had in the first place (Allison 2013, p. 5). Our respondents grow up without the expectation that employers will care for them. Standing (2011) refers to the people affected by these trends as ‘the precariat’.
4. The European Research Council’s ethics board demanded that if respondents were younger than 18, we obtain their parents’ permission for them to engage in the research. This was not practical, as many youth live away from their families We also did not consider it ethically appropriate to do so, given the nature of some of their chemical practices and our commitment to anonymity. However, the European Research Council’s ethics committee demanded that we did not analyze the fieldnotes that concerned youth below the age of 18.
5. This interview tool can be found on the ChemicalYouth website (chemicalyouth.org) under the methods section.
6. Because of the risks related to the illegal use of drugs in most of our study sites, the ChemicalYouth project adopted anonymity procedures. Informants were assured that their participation was both completely voluntary and anonymous. Before conducting any interviews in the GrandTours, PhD field researchers and junior researchers in all four countries were trained by their respective PI’s in keeping and maintaining a vigorous commitment to the anonymity of participants. For example, interviewers made sure to inform participants that any and all identifying details that they gave (such as name, address, date of birth, place of birth) would be removed from their transcripts, that a pseudonym would be given, and that interviews would take place in public spaces that allowed for private discussions. Furthermore, researchers were trained on using a system of acronyms to title their interview transcripts and other documents that did not contain the name of the respondent. Anonymity was of particular importance in our focused ethnography of an online forum of drug users reporting and sharing their experience with new psychoactive substances (see Berning and Hardon 2016). In order to safely report on their online data, and not have their quotes be traceable on search engines, their online pseudonyms were pseudonymized again and the names of the fora were not revealed. These safeguards were approved by an independent ethics advisor. One exception to adhering to our anonymity guidelines is the focused ethnographies discussed in chapter seven with supplement creator and vitamin shop owner. Upon consideration, it was concluded that they were well-known internet personalities, therefore their names were easily identifiable. Moreover, the products that they sell are legal and thus sharing their real identity or their company did not pose a risk as it would have, had they been selling illegal products.
